# Commuter Mobility and the Spread of Infectious Diseases: Application to Influenza in France

**DOI:** 10.1371/journal.pone.0083002

**Published:** 2014-01-09

**Authors:** Segolene Charaudeau, Khashayar Pakdaman, Pierre-Yves Boëlle

**Affiliations:** 1 INSERM, UMR S 707, Paris, France; 2 Université Pierre et Marie Curie - Paris 6, Paris, France; 3 Institut Jacques Monod, Paris, France; 4 Université Denis Diderot, Paris, France; Northeastern University, United States of America

## Abstract

Commuting data is increasingly used to describe population mobility in epidemic models. However, there is little evidence that the spatial spread of observed epidemics agrees with commuting. Here, using data from 25 epidemics for influenza-like illness in France (ILI) as seen by the Sentinelles network, we show that commuting volume is highly correlated with the spread of ILI. Next, we provide a systematic analysis of the spread of epidemics using commuting data in a mathematical model. We extract typical paths in the initial spread, related to the organization of the commuting network. These findings suggest that an alternative geographic distribution of GP accross France to the current one could be proposed. Finally, we show that change in commuting according to age (school or work commuting) impacts epidemic spread, and should be taken into account in realistic models.

## Introduction

The multi-scale network of social interactions [1], [2] makes rapid dissemination of transmissible diseases possible, as illustrated recently by pandemic A/H1N1 2009 influenza and SARS [3], [4]. In this context, predicting the efficacy of public health interventions requires the identification of the most relevant factors for dissemination [4]–[5]. For instance, international air travel was found to provide good prediction for the worldwide spread of SARS and influenza A/H1N1 2009 [3], [4]; it was however shown that intervention on the global air traffic would be of limited efficacy [6]. At a more local scale, air travel is less relevant and other types of movement must be taken into account. Commuting, i.e. daily movements from residence to work or school, has been widely used to describe spatial mobility in models, using exhaustive datasets [7], [8] or gravity models [9], [10].

Except for a report on the correlation between influenza epidemic peak timing and inter-states commuting in the USA [9], whether commuting may explain the spatial spread of epidemics has been little studied. Influenza like illness (ILI) incidence time series, as monitored by the Sentinelles network since 1984 in France, provide data at a high spatial resolution (NUTS3) that can be used in this respect (http://www.sentiweb.org). These data, unique in duration and spatial resolution, helped elucidate long sought questions like the impact of school closure during epidemics [11] and to validate model predictions for pandemic flu [12]. Commuting data based on the census of the population is also available at an even finer scale.

Using these two databases we first analyzed how commuting data relates to disease spread at a local level. We then examind the underlying mechanisms of propagation using an epidemic model derived from commuting networks An indicator based on the similarity of epidemic courses in excess of random movements was developed. Finally, we investigated how age differences in commuting networks, i.e. to school or to work, led to changes in the spatial spread of diseases.

## Materials and Methods

### Data

#### Sentinelles data

**Figure 1:**
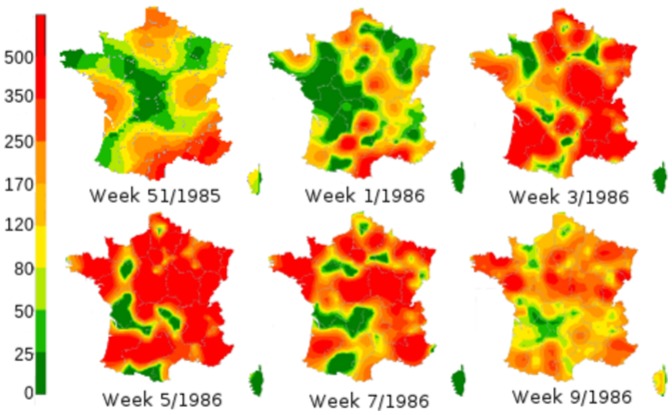
Spatial spread of influenza like illness in France. Incidence for 100000 inhabitants as monitored by the Sentinelles network during season 1985–1986. Maps are 2 weeks apart.

The Sentinelles network [13] is comprised of over thirteen hundred general physicians (GPs), accounting for approximately 2% of the total number of French GPs. They report the number of observed influenza-like illness cases on a regular basis, using a standardized case definition (more than 39C fever with myalgia and respiratory syndromes). We used the data of 26 consecutive seasonal influenza epidemics, from 1985 to 2010 (Figure 1). The data was obtained on a weekly basis at the NUTS3 (‘department’) level. There are 95 NUTS3 areas in France. To jointly analyse multi-year epidemics, we defined each year week 0 as the national epidemic peak, and considered 15 weeks of data before and after this date.

#### Demography and commuting

We used the data collected in the 1999 census data in France. All data were obtained at the LAU1 level, that we refer to as ‘district’ afterwards. There are 3704 districts in France. In each district, the population was split into 5 age classes : less than 3 years old; 3 to 10; 11 to 18; 18 to 65 and more than 65. These categories were retained to capture large changes in mixing groups due to schooling (3–10 and 11–18) and work (18–65). The frequency of each age class was obtained from census data in each district, as well as the percentage of population with a professional occupation. We also computed the average number of contacts of an individual of age 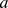 with members of the same household of age 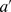 in each district, denoted by 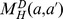 in district 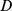.

The commuting dataset, derived from census data, contains the movements of more than 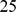 millions of adults and 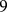 millions of children. Commuting frequencies between districts were computed as a matrix 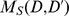 for school-based commuting and 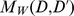 for work-based commuting, where 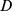 stands for the district of residence and 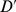 for the district of destination. The matrices were normalized by rows, yielding the percentage of the population of the district of residence commuting to the district of destination; for example 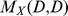 was the percentage of people remaining in their district of residence for school or work.

We identified communities using the weighted ‘Louvain’ algorithm [14]. This algorithm clusters nodes by maximizing the weight of links within each cluster while minimizing that between clusters. The communities identified with the school commuting network and the work commuting network were compared with the Jaccard index, which compares 2 clusterings by measuring the number of district pairs that are gathered together in both clusterings over the number of comparable district pairs (a pair of districts is considered comparable if the 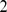 units belong to the same community in at least one clustering).

### Disease transmission model

#### Natural history of influenza infection

The natural history of influenza infection was described as a 4 stage SEIR process: individuals were first susceptible to the disease (stage S), then latent (infected but not infectious yet; stage E), infectious (stage I) and finally recovered and removed from transmission (stage R). We simulated transmission using the generation time distribution, i.e. the time from infection in a primary case to infection in a secondary case, as in Mills et al. [15]. For all asymptomatic cases and symptomatic cases within households, the generation time distribution was modelled by a gamma distribution with mean 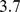 days and standard deviation 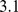 days. For symptomatic cases in the community, the generation time was gamma distributed with mean 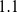 days and standard deviation 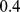 day [16]. These differences account for the reduced time spent in the community, school or workplace by symptomatic cases. We assumed an initial percentage of susceptibility of 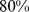, irrespective of age.

#### Transmission

A discrete time (time step 0.2 days) deterministic transmission model was implemented. We assumed that only professionally active individuals in age class 18–65 would commute to work, and that all children aged 3 to 18 attended and commuted to school. School-based commuting matrices were the same in age classes 3–10 and 11–18. No births and deaths were considered during the time of simulation, nor any change in place of residence or of destination.

At each time step, the number of incident cases 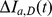 in age class 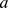 and district 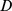 was computed as 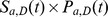 where 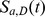 was the number of susceptible individuals and 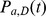 the probability of infection. The probability of infection was calculated according to the following equation:

(1).
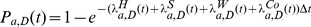
(1)where 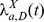 was the force of infection exerted on an individual of age 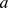 in district 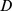 from place 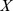.

Household based force of infection was computed using the age-specific average number of contacts in the household. More precisely, the force of infection was proportional to the density of infected contacts among household members as follows (2) :
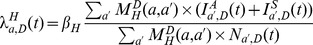
(2)where 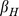 was the pairwise rate of contact leading to transmission in the household. 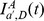 and 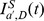 were respectively the number of asymptomatic and symptomatic incident cases, which were considered equally able to transmit the infection.

For school-based (X = S) and workplace-based (X = W) force of infections, we used a similar approach, computing the expected density of infection among contacts as (3):
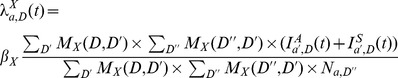
(3)here 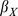 was the pairwise rate of contact leading to transmission. Using this formulation, the contacts in place 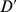 are counted with all people effectively commuting to this place, from place 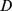 as well as from all places 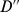 directly connected to 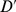.

For community based transmission, the force of infection was computed using the same principle as above by (4).
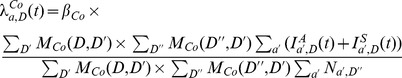
(4)where the sum was on all districts 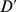 sharing a border with district 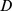. To take into account the different behavior of people during day and night, we considered that individuals were only commuting during the day, and staying at home during the night. Therefore, we considered that individuals could only interact within their households at night.

We calibrated transmission parameters 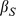, 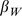, 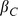 and 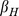 so that simulated epidemics had durations and attack rates consistent with observed epidemics (see http://www.sentiweb.org). More precisely, in the Sentinelles network, a typical epidemic starts when incidence increases over 150 cases/100000 per week, and remains above this threshold for approximately 10 weeks; the cumulated excess cases during this period ranges between 2 and 8 percent of the population. We selected parameters with which the duration with an incidence larger than 150/100000 was 10 weeks, and the excess cumulated cases was 5.5% of the population. Several sets of 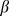 values were still possible, and we finally selected values so that one half of the cases were due to school or work transmission (respectively 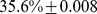 and 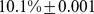), and the other half to local transmission (household and community, respectively 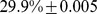 and 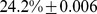 of transmission). This repartition compared with other choices reported in [17] and [7], although we put a little more weight on school/work transmission. Using these parameters, the initial exponential growth coefficient of the epidemic was 0.75 log(person)/week, in the same range as those observed during the last 25 epidemic seasons in France (0.5 to 1.0).

### Statistical analysis of data and results

#### Spatial auto-correlation analysis

Moran's I statistic [18] was used to evaluate the spatial auto-correlation of ILI incidence data. Moran's I was calculated by:
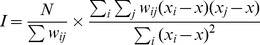
(5)where 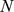 is the number of spatial units, 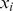 the incidence observed in unit 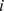 and 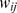 the spatial weight of the link between 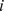 and 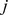. Moran's I ranges between −1 and 1, with negative values indicating negative correlation among neighbors, while positive values indicate positive correlation. To assess whether commuting agreed with spatial incidence, we computed the 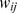 as the size of the population commuting between 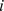 and 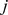 [19].

Moran's I was computed for each week before and after epidemic peaks, and averaged, week-wise. The same procedure was repeated 1000 times using random permutations to calculate p-values. To test for the specific role of the commuting network as opposed to commuting distance only, we compared these indices with those obtained using random commuting networks, where the distribution of distance travelled was kept the same as in the original data, but commuting trips were chosen at random in any direction. We repeated the above calculation for 100 such random networks.

We also used Mantel's test as described in [9]. The correlation between incidence time series was first calculated for all pairs of departments, then compared with the flows (ingoing and outgoing) between departments.

In all cases, permutation tests were used to calculate P-values.

#### Overlap between epidemics

We used the overlap measure introduced in Colizza [20], that takes into account the similarity in spatial spread, as well as in total incidence. Values close to 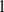 indicate similar incidence in all places at a given time, while values of 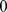 correspond with little overlap. In all cases, epidemics were started with one infected children in a single district. The overlap between two epidemics, started in districts 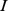 and 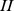, was calculated as
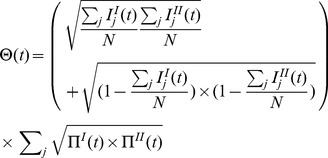
(6)where 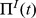 described the geographical distribution of incidence among districts at time step 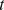 in epidemic 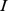, and 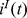 was the incidence per population at time 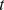. The overlap measurement is for a given time 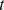. Irrespective of the starting places, the overlap measure always grew to 1 with time.

**Figure 2:**
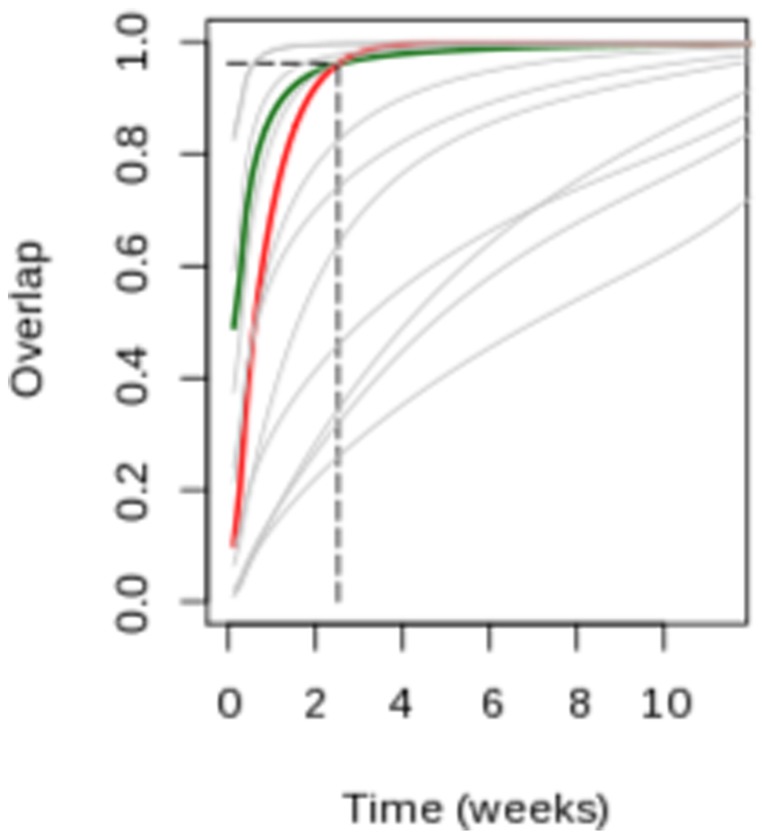
Measuring similarity in spread above randomness 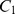. Lines correspond with overlap measures for a given pair of district at different times after introduction of a single infected. For a particular pair (green line), we also present the overlap measure obtained using reshuffled networks for the same pair (red line). Criterion 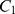 was defined as the time when the green line crossed the red line.

For each pair of districts in France, we aimed to identify up to what date after first introduction epidemics grew more similarly than expected if commuting was at random. This is measured by criterion, 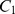 that we computed as follows. First, the commuting networks were reshuffled, by permuting, at random, the destinations in the original network. This procedure retained the distribution of degrees in incoming and outgoing links, but randomized the destinations all over France, implementing a random commuting network. Then, epidemics were simulated starting from the same pair of districts using the reshuffled networks. The “above randomness” part was computed as the time during which the overlap of the epidemics simulated using the original networks was larger than that with the reshuffled networks (Figure 2). Large values of 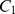 indicated that the two epidemics looked alike for a long time.

#### Sensitivity analysis

To test the sensitivity of the model to the proportion of infections occuring in each context, we performed 100 simulations with a set of parameters, for which 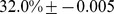 of transmission occured at home, 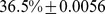 at school or work and 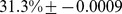 in the community, starting from randomly selected districts. Overlap was used to compare these simulations to the former ones.

An analysis of sensitivity was also performed to test the impact of the hypothesis that adults asymptomatic individuals had a reduced generation time, by simulating 100 outbreaks with a random initial case where only children would have it. As before, overlap was used to compare the simulations to the former ones.

The sensitivity of the results to the proportion of adults initially immunized was also tested, simulating 100 outbreaks intitialized in randomly chosen districts with different rate of immunity (0, 10, 20, 30, 40, 50, 60 and 70%). Simulations were compared to outbreaks generated with a 80% rate of immunity for adults using overlap.

## Results

### Commuting networks

**Figure 3:**
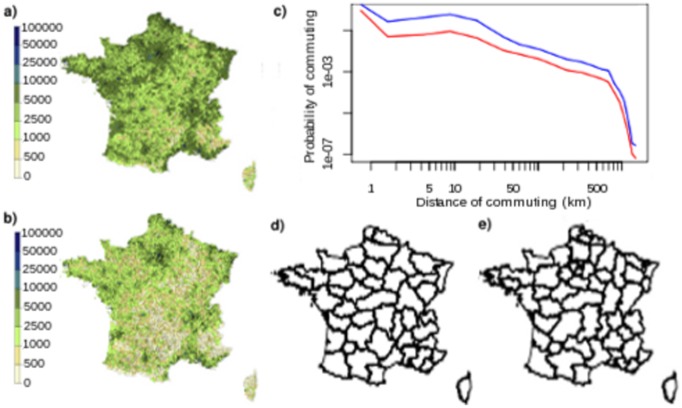
Commuter mobility in France. (a,b)Total number of individuals leaving each district via work commuting (a) and school commuting (b). (c) Proportion of commuters and travelled distance in the school network (red) and the work network (green). (d,e) Clusters identified in the work (d) and schoool (e) commuting networks.

Workers from one district commuted on average to 133 other districts, and school aged children to an average 75 destinations (Figure 3-a,b). The average commuting distance was 14.8 km and 12.4 km for work and school, with 15% of workers commuting outside their department, but only 6.7% for children (Figure 3-c). Long distance travel (>100 km away) was however as common for work and school (1.5% of the cases).

The diameter (i.e. the longest minimal path from one place to the other) of the commuting network was 3 for work and 4 for school.

The importance of short-distance commuting also showed in the communities found by clustering (Figure 3-d,e). Indeed, all communities were constituted of adjacent districts, although this is not a constraint of the method. The Jaccard index for the work and school communities was 0.519, showing that approximately half the districts belonged to the same community in both the work and school networks. The differences arose for the most part from places along the borders between clusters. The work network produced less communities than the school network, especially in the Paris region, highlighting the more local structure of school commuting.

### Commuting and observed epidemics in France

**Figure 4:**
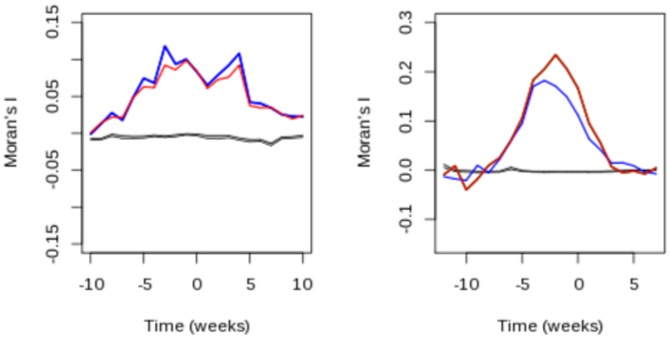
Autocorrelation in incidence for observed and simulated epidemics. (a) Mean value of Moran's Index computed on the 26 epidemics from the Sentinelles network, and (b) on 100 simulated epidemics. In each case, the blue line uses work commuting based weights or school (red line). Gray areas corresponds to the 95% expected values when no autocorrelation is present.

In the 26 epidemics observed in the Sentinelles network, the spatial autocorrelation computed with weights derived from school and work commuting was significantly greater than 0. In other words, incidence increased synchronously in strongly linked areas. Moran's I was significantly greater than 0 (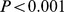) as soon as 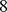 weeks before the national peak and remained greater than 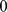 up to 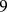 weeks afterwards(Figure 4-a), with maximum value 1 to 3 weeks before the date of the national peak. The magnitude of Moran's I was approximately the same with all spatial weights.

Likewise, Mantel's test performed with weights matrix derived from school and work commuting was positive (Mantel's correlation being equal to 0.069 for work commuting and 0.060 for school commuting), confirming the existence of a spatial auto-correlation linked to commuting movements (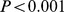).

### Commuting and simulated epidemics

Simulated epidemics started from different places were all similar in timing and incidence at the national level. Moran's I analysis exhibited the same behavior as in the observed epidemics (Figure 4-b) and was significantly positive using all weight matrices. Here again, the index increased as the epidemic spread and was the largest shortly before the date of national peak.

As for observed epidemics, Mantel's test was found to be positive for simulated epidemics (mantel correlation was equal to 0.106 with work commuting and 0.121 with school commuting).

#### Overlap in initial epidemic spread

Irrespective of the starting district, national incidence was very similar over the course of the epidemic. Even if the national incidence were similar, overlap changed depending on the pair of districts considered. Initial overlap was very variable using the observed commuting network, but always increased to 1 with time. Remarkably, the overlap in epidemics using reshuffled networks was also large, and quickly increased to 1 as well.

The excess in overlap, as measured by criterion 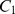, ranged from 0 to more than 180. The first case arose for epidemics started from distant places, with 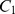 increasing in neighboring districts. There was a large negative correlation between 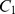 and distance (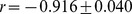, Spearman correlation). Almost all district pairs more than 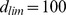 km away had 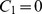, in other words epidemics started from districts more than 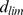 km away showed little resemblance in initial spread.

On the contrary, 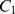 increased when the two starting districts were closer, indicating spread on common paths. However, the variance of 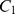 was large, even at small distances, indicating that distance was not the only condition for similar spread. For example, 2 epidemics started in districts less than 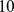 km away could be less similar than 2 epidemics started more than 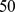 km away; and epidemics started from less than 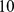 km away could have a very similar spread or quickly diverge depending on the pair of districts considered.

We found that the correlation between 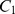 and the proportion of commuters between districts was also large (r = 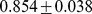), and that both distance and volume contributed to the value of 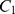: The partial correlation between 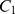 and the proportion of commuters, conditional on distance, was 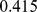. The coefficient of determination of distance and proportion of commuters on 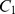 was large: 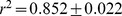.

**Figure 5:**
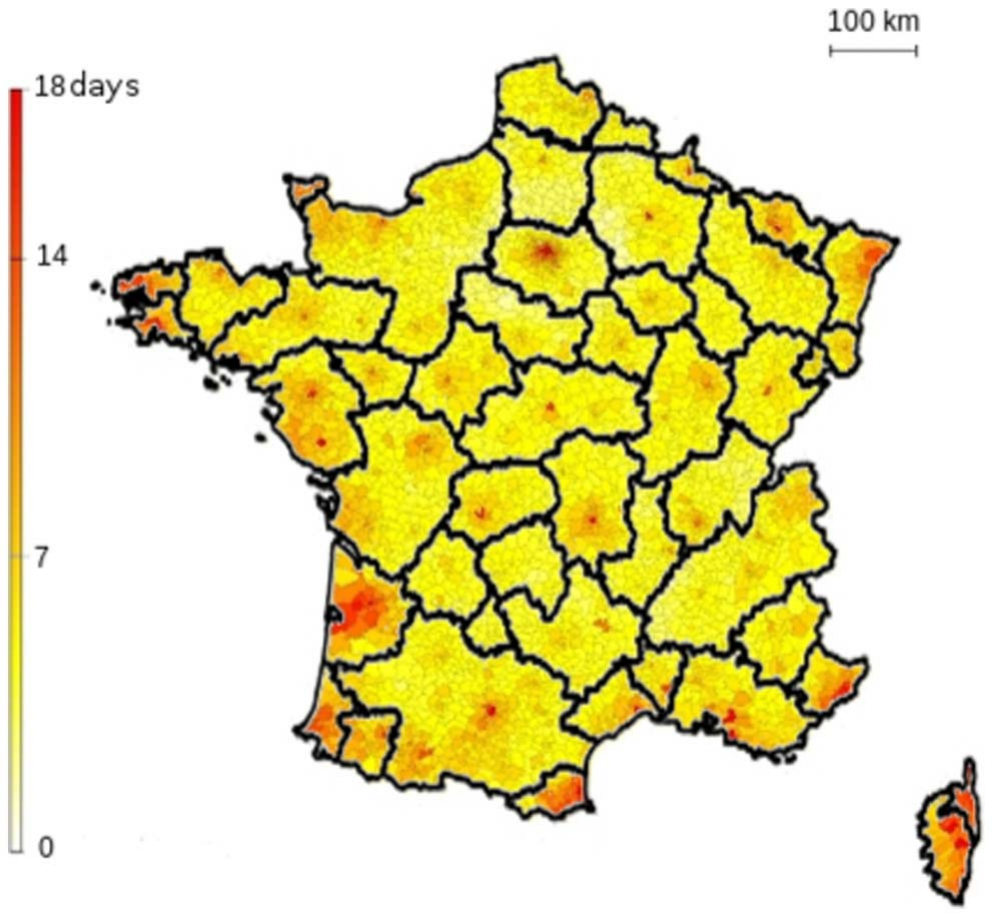
Typical pathways according to initial infective location. For each district, 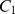 values were averaged over all neighbors less than 100 km away. Basins of attraction were identified by clustering.

To get a picture of initial common paths of spread, we averaged the value of 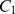, in each district, over all neighbors less than 100 km away. A large value indicated common initial paths in all epidemics started in close neighbors. Figure 5 illustrates these preferential paths, as evidenced by large values of average 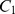 in several places. Among the districts having the largest values of 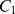, many were large French cities, like Paris, Toulouse or Marseille: 30 of the 50 largest French cities were among those with the largest 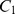 values. Other districts with large average 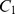 were found as suburban cities close to large cities; and some in coastal or border districts. Overall, there was a large correlation between average 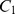 and the number of inhabitants in each district (r = 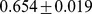).

Based on the average 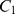 value, we obtained 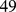 communities based on Louvain clustering (Figure 5). Most of these clusters included one or two very populated French cities, for which the average value of 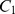 was the highest of the community. 33 clusters included one of the 50 largest French cities and 5 other included a city less important in size, but large relative to its neighboring districts. Other large French cities were included in previous clusters, as they were strongly connected to a large city (Aix, for example, 22nd biggest city in France, was aggregated with Marseille, 2nd most populated city, which is both close and well connected to it). 6 of the remaining clusters did not include major French cities and corresponded with sparsely populated areas. Finally, coastal or border districts tended to cluster together on a geographical basis.

### Age dependent commuting networks

Commuting for work and school created two layers of mixing that could lead to differences in the spatial spread. Indeed, the distance traveled to work was larger, suggesting increased dissemination, but transmission in children is typically larger and could take precedence on transmission by adults. We therefore simulated the spread of epidemics in models where either commuters for school or work remained in their place of residence, with the same number of contacts.

**Figure 6:**
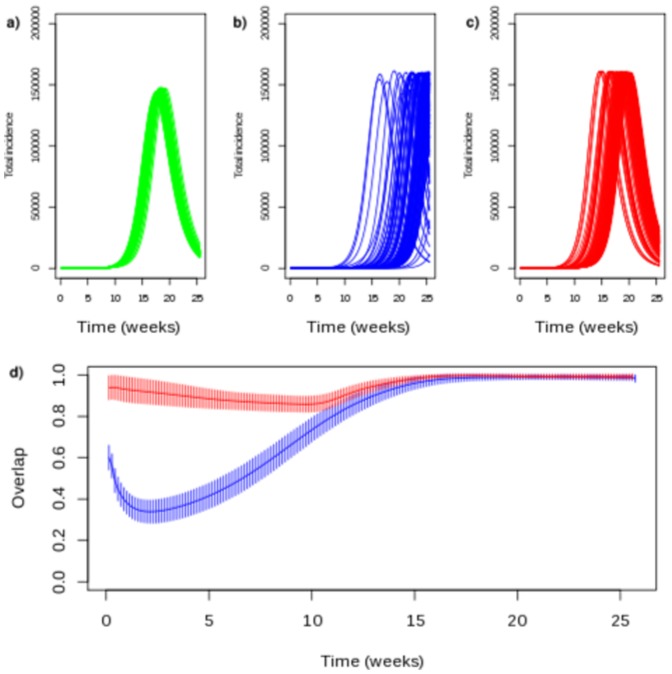
School and work commuting networks and the spatial spread of epidemics. (a,b,c) ILI epidemic curves using all commuting networks (a), only work commuting (b) and only school commuting (c). Epidemics were started form 1000 randomly chosen districts. (d) Overlap between epidemics using work (blue curve) or school commuting (red curve).

Epidemics were started from 100 random districts with the 3 possibilities : commuting to work and school, only to school or only to work (Figure 6-a,b,c). Epidemics simulated with the two commuting reached a national peak in a narrow time window, the time of peak slightly depending on the size of district of departure population (correlation 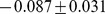) or on the number of commuters sent by the district of departure in the school and the work network (correlation were respectively 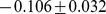 and 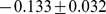). The final attack rate was not influenced by the district of departure. The spread of epidemics simulated with only one type of commuting was more variable, with an increased range of time to the national peak.

Not unexpectedly, ignoring one commuting network led to epidemics that spread less rapidly. The peak of epidemics simulated with school commuting were on average delayed by 2 weeks, although with large variability. For some simulations, the propagation was faster when only school commuting was present, but this was independent of the district of departure (correlation of delay with district population : 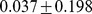; correlation with the number of children commuting from the district : 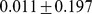). The impact was more important for epidemics simulated with work commuting, which were more delayed, and with highest variability.

**Figure 7:**
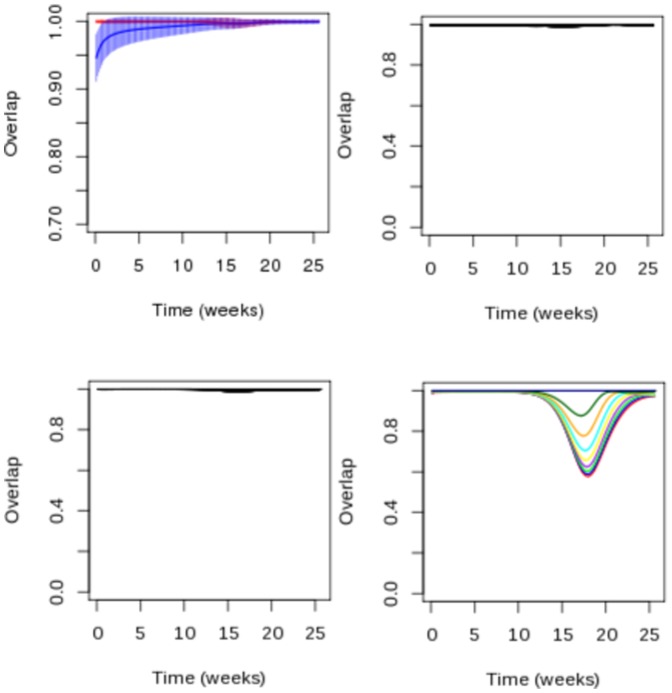
Sensitivity analysis. Overlap between epidemics simulated with first model and epidemics propagating only by school (red) or work (blue) commuting (a), with epidemics for which asymptomatic adults do not have a reuced genration time (b), with epidemics simulated with different parameters of transmission (c). (d) Overlap between epidemics in which 80% of adults are susceptible with epidemics with different rates of susceptibility.

Finally, simulated outbreaks where all commuters followed the same commuting pattern, either school or work, were much in line with the results above. Overlap with original simulations was almost perfect when using only the school network but differed markedly from the start when using only work commuting (w Figure 7 -a).

#### Sensitivity analysis

The overlap between simulations with different rates of contacts and the original simulations started in the same district was very large (Figure 7 -b) as 95% of overlap values ranged between 0.9929 and 0.9998 through the entire course of the epidemic. This indicates that the spread of the epidemic was very similar in both cases and that our results regarding to how networks shape the initial spread were robust to this modification.

Similarly, the overlap between epidemics with a reduced generation time for symptomatic adults and without was very large (Figure 7-c) with 95% of overlap values ranging between 0.9931 and 0.9999 during the whole course of the epidemics. This showed that the results regarding initial spread of the disease was robust to this assumption.

The overlap between simulations with 80% of susceptible adults and other percentages of immunization decreased with the rate of susceptibility of adults (Figure 7-d).

## Discussion

Our analysis showed that commuting data determines the spread of influenza in modern populations, as evidenced by the large autocorrelation in observed ILI incidence in regions connected by commuting. Building on this observation, we provided an in depth study of the consequences of mobility as described by commuting in the initial spread of epidemics, showing how to identify preferential paths in a densely connected territory. Last, we showed that age specific heterogeneity in commuting leads to different patterns of spread, depending on the age category the most involved in transmission.

The spatial structure of epidemics in France was manifest according to the change in Moran's index over time. The index increased up to a maximum just before the national epidemic peak, and decreased afterwards. This spatial structure was hinted at by the non random structure of spatial incidence pointed out by Bonabeau et al. [21] and the decreasing correlation with distance found by Crepey et al. [22]. However, neither of these studies linked these observations with human mobility. Here, we showed that these properties could be explained by commuting, strengthening the case for using commuting data to model the spatial spread of diseases at a regional scale. We measured the correlation between incidence and commuting using Moran's I and Mantel's test. These provide complementary information regarding the association of commuting with spatial disease spread. Indeed, Moran's I compares magnitudes in connected regions, while Mantel's test is more sensitive to the timing of the peaks between epidemics. As in Viboud [9], Mantel's test supported the hypothesis of correlation between epidemic spread and commuting volume. Our conclusions are further supported by the fact that in the simulated epidemics, Moran's I and Mantel's test displayed the same pattern as for observed epidemics.

In our systematic exploration of the model dynamics, a three stages scenario for the spread of epidemics emerged. The first stage followed introduction of an infected individual in the population. The lack of large 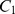 value for districts more than 100 km apart reflected the spatial scale of this first phase, and the large variance in 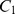 values evidenced the strong dependence on the initial location for initial spread. During this stage, transmission occurred in the initial community and its proximal districts over a few weeks. It ended when infection reached an amplifier district. This was illustrated by the existence of districts with a large average 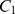 value, showing that these places produced epidemics that were very similar to those started around. The second stage saw the spread from the first amplifier district to other districts at a longer range, via long distance links. In this second stage, it was mostly large cities that were attained all over the territory. The last stage started with the spread around large cities, but quickly led to transportation of cases both locally and globally, yielding the national epidemics. Importantly, this structure arose from the features of observed commuting data. One of the challenges was to be able to identify the amplifier nodes and their basins of attraction, and the downstream propagation paths directly from such data. This is where the methods introduced in our paper are of broader interest.

We used the raw commuting data from the census, instead of a smoothed version based on a gravity model [9], [23], [24]. As our data was exhaustive, it was not necessary to use modelling in the first place. Using raw data leads to more heterogeneity in commuting links, given different districts at the same distance and with the same population may not receive the same number of commuters. It may also lead to results that are very dependent on the reported mobility, which captures only a part of human mobility. Allowing individuals to mix in a local community (district and close neighbors) was a way to keep the particular features of the commuting data, while allowing for inaccuracies or random moves not measured in commuting. We also chose to differentiate school and work commuting, when most metapopulation models either ignore school commuting [9], [23] or assume the same rate of contact between individuals in the 2 contexts [24]. In our simulations, we found that the interactions of the two networks tended to homogenize epidemic curves, irrespective of the starting location. Indeed, the timing of the peak was in a very limited range, irrespective of the starting place. With our choice of parameters, the spatial spread of the disease was driven more strongly by school commuting than by work commuting: removing the work network affected less overall transmission than the converse. The prominence of the school network is likely a consequence of our assumption that over 40% of all transmissions occurred in school. However, this analysis shows that differences in commuting networks could lead to changes in spatial spread. For example, it was reported that school holidays mostly affected how quick a disease would spread [25], [26], but this result did not take into account differences between work and school commuting. Our results show that closing schools may also affect preferential paths of spread.

Seeding epidemics with only one case, as we did in the systematic analysis, is presumably not very realistic. Indeed, real epidemics may be seeded by repeated introductions from abroad over a few weeks. We however selected this simple seeding pattern to study systematically the influence of the initial place of introduction, as it allowed a rather simple way to compare epidemic courses through their overlap. This type of seeding likely reduces noise and leads to increased spatial autocorrelation, as noted in Figure 4.

Thanks to the systematic search for locations having large similarity with others, we identified preferential paths for epidemic spread due to human mobility. Clustering districts according to the average 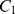 measure allowed to define clusters showing the ‘basin of attraction’ for these preferential paths, as shown in Figure 5. Most clusters were centered around an important city of the area, which may not be highly populated compared to other cities, but was relatively important compared to neighboring places. The role of such places must be studied further in the context of epidemiologic surveillance. Indeed, it suggests that to capture a new epidemic, it would be interesting to have at least a GP in each cluster. It must be studied whether this would be more effective than allocating surveillance based on population coverage [27]. Moreover, as the behavior of epidemics from any district in a cluster tends to resemble the behavior from a central city, focusing on the main cities identified in the study could lead to the optimal use of GPs for surveillance.
